# High prevalence of sub-microscopic infections in Colombia

**DOI:** 10.1186/s12936-015-0711-6

**Published:** 2015-05-15

**Authors:** Andres F Vallejo, Pablo E Chaparro, Yoldy Benavides, Álvaro Álvarez, Juan Pablo Quintero, Julio Padilla, Myriam Arévalo-Herrera, Sócrates Herrera

**Affiliations:** Malaria Vaccine and Drug Development Centre, Cali, Colombia; National Institute of Health of Colombia, Bogotá, Colombia; Caucaseco Scientific Research Center, Cali, Colombia; Ministry of Health and Social Protection of Colombia, Bogotá, Colombia; Latin American Center for Malaria Research, Cali, Colombia; School of Health, Universidad del Valle, Cali, Colombia

## Abstract

**Background:**

Malaria transmission in Latin America is typically characterized as hypo-endemic and unstable with ~170 million inhabitants at risk of malaria infection. Although Colombia has witnessed an important decrease in malaria transmission, the disease remains a public health problem with an estimated ~10 million people currently living in areas with malaria risk and ~61,000 cases reported in 2012. This study aimed to establish the malaria prevalence in three endemic regions of Colombia to aid in designing new interventions for malaria elimination.

**Methods:**

A cross-sectional survey was conducted in three regions of Colombia with different malaria epidemiological profiles: Tierralta (Ta), Tumaco (Tu) and Buenaventura (Bv). The Annual Parasite Index (API) was 10.7, 6.9 and 3.1, respectively. Participants were asked to respond to a sociodemographic questionnaire and then were bled to determine the Duffy genotype and the prevalence of malaria infection by microscopy and quantitative real-time PCR (qPCR).

**Results:**

The study was conducted between October 2011 and January 2012. Eight sentinel sites with 1,169 subjects from 267 households were included. The overall prevalence of sub-microscopic infections measured by thick blood smear (TBS) was 0.3% (n = 4) whereas by qPCR it was 9.7% (n = 113), with a greater proportion (13%) in 40-50 years old individuals. Furthermore, different regions displayed different prevalence of sub-microscopic infections: Bv 12%, Ta 15%, and Tu 4%. From these 113 samples (qPCR), 74% were positive for *P. vivax* and 22% for *P. falciparum*, and 4% were mixed infections, which correlates to the overall parasite prevalence in Colombia. This study showed that in the southern Pacific coast of Colombia (Bv and Tu), around 56% of the population have a Duffy-negative genotype, compared to the northern region (Ta) where the percentage of Duffy-negative genotype is around 3%.

**Conclusions:**

Sub-microscopic infections are prevalent across different regions in Colombia, particularly in areas with relatively low transmission intensity. The poor microscopy results suggest the need for more sensitive diagnostic tools for detection of sub-microscopic infections. This study underscores the importance of conducting active case surveillance to more accurately determine malaria incidence, and highlights the need for updating the malaria guidelines to track and treat sub-microscopic malaria infections.

## Background

The burden of malaria has declined in recent years across a number of countries as a result of increasing funding and political commitment, implementation of new anti-malarial treatments, better access to diagnostics, and the extended use of insecticide-treated bed nets and indoor residual spraying [1-3]. This reduction represents a challenge for malaria control programmes to ensure sustainability and the impact of routine prevention and control measures, which should be adapted to the new epidemiological settings. Since in low transmission settings control interventions are likely to be less effective [1]; as transmission declines national malaria control programmes (NMCPs) must adapt and introduce necessary changes through the development and adoption of novel strategies and methods. Operational research contributes to this goal by providing evidence base for decision-making.

Clinical manifestation of *Plasmodium* infection varies from asymptomatic to severe and fatal malaria. Continuous exposure to *Plasmodium* parasites leads to clinical immunity and asymptomatic carriers [4,5]. In addition, asymptomatic cases provide a fundamental reservoir of parasites contributing to malaria transmission. A better understanding of the role asymptomatic infections play in transmission is essential for adapting and improving the responsiveness of NMCPs and improving malaria elimination programmes [6-9].

Although microscopic diagnosis continues to be the ‘gold standard’ technique for malaria, including certification of malaria elimination [10], molecular diagnostic methods currently available would greatly contribute to assessing the malaria burden in hypo-endemic regions. However, scaling up molecular surveillance methods is still work in progress. Rapid diagnostic tests (RDTs) that are easy to handle and affordable significantly contribute to accelerate case detection but they have equivalent sensitivity/specificity to microscopy. Other techniques such as quantitative real time polymerase chain reaction (qPCR) are more sensitive [11,12], and more appropriate when malaria transmission approaches zero cases. Currently, the fact that these asymptomatic infections are largely very low density and not detected with the operative diagnosis methods (thick smears and RDTs), represents a great hurdle for malaria elimination [13,14]. In order to achieve successful elimination, detection of all parasite carriers by active case detection and then treatment of all infections must be considered to interrupt malaria transmission in endemic areas. The identification and management of sub-microscopic carriers has become a new and increasingly important challenge for malaria control programmes [15]. The treatment of all infections as part of routine surveillance strategies has the potential to contribute significantly to the reduction of malaria in endemic regions.

Although asymptomatic infections have not been extensively studied in Colombia, there is growing evidence that sub-clinical infections are more common in the region than previously thought [16,17]. This study aimed to determine the malaria prevalence in three malaria-endemic regions of Colombia.

## Methods

### Study sites

Three areas were selected in Colombia. Sentinel sites in each of these areas were selected based on the annual parasite index (API), accessibility and safety.

Tierralta is a municipality located in the Department of Cordoba in the northwestern part of Colombia with ~90,000 inhabitants composed of ~2% indigenous groups and 44.4% rural population. The Annual Parasite Index (API) in 2011 was 10.7. The predominant malaria parasite species in this region is *Plasmodium vivax* (82.4%) followed by *Plasmodium falciparum* (17.4%) and mixed malaria infections (0.2%) [18]. The selected sentinel sites in this region were Tuis Tuis and La Unión.

Tumaco is located in the Department of Nariño in the southwest of the country near the Ecuador border. It has a population of ^~^187,084 inhabitants composed mainly of Afro-descendants (88%). The API in 2011 was 6.9. The predominant malaria parasite species in the region is *P. falciparum* (79.2%) followed by *P. vivax* (20.8%) [18]*.* The sentinel sites selected in this region were Robles, Candelillas and Bucheli.

Buenaventura is located on the Pacific coast of the Valle Department in the west of Colombia, with ~350,000 inhabitants who are predominantly Afro-descendants (72.4%). The API in 2011 was 3.1. The predominant malaria parasite species in the region is *P. vivax* (85%) followed by *P. falciparum* (15%) [18]. The sentinel sites selected in this region were Punta Soldado, Zacarias and La Delfina.

### Study design

The study was a cross-sectional survey in three malaria-endemic regions of Colombia, which is one of the countries in the network of the Latin American Center for Research on Malaria (CLAIM) established with the National Institutes of Health (NIH) funding in 2010. The study was conducted between October 2011 and January 2012 in eight sentinel sites.

The study was developed in two steps: first, a population census was performed with door-to-door visits and the help of community leaders. The number of houses, people/house, age, and sex were recorded. Based on this information, a probabilistic sample was calculated for each sentinel site with a confidence level of 95%, and error of 2% and an estimated prevalence of 2%. Second, a set of houses was randomly selected, and every person from the household present at the moment of sampling answered a questionnaire regarding symptoms and epidemiologically relevant information. Males and females of all ages were included. No others visits were made.

### Samples

Blood samples were collected by finger prick on filter paper for malaria parasite DNA isolation and qPCR diagnosis. For Duffy genotype determination, a blood sample of 500 μL was collected by venipuncture from every subject and stored in EDTA containing tubes. Samples were handled as potential biohazards and all laboratory staff strictly followed bio-safety standardized procedures.

### Parasite detection

#### Thick blood smears

Approximately 100 μL of blood were collected by finger prick and both thick and thin smears were prepared and stained by Giemsa method for malaria diagnosis. Each slide was examined separately by two experienced microscopists, recording species and densities of sexual and asexual forms. In positive films, parasite species were identified and density recorded as the number of parasites per 200 white blood cells (WBC). Two-hundred high-power fields of the thick films examined at 1,000× magnification were read before recording a negative result. Inter-observer discordances were solved with the reading of a third microscopist. Subjects were considered positive for malaria only if two of the three readings were positive.

### PCR methods

#### qPCR

Assays were performed as described previously [19,20] with minor modifications. DNA was extracted from three Ø 3 mm filter paper punches, equivalent to approximately 15 μL blood, using the PureLink Genomic DNA kit. DNA was eluted in 60 μL of buffer. The amount of DNA in each well was adjusted to be equivalent to 1 μL of whole blood (4 μL of DNA). Standard *P. falciparum* and *P. vivax* DNA positive and negative controls were used in each batch of tests, including the extraction of both negative and inhibition control. A sample was considered negative if there was no increase in the fluorescent signal after a minimum of 40 cycles. Parasitaemia quantification was performed using a parasite-specific standard curve obtained by diluting *P. vivax* and *P. falciparum* field isolates to densities of 20000, 2000, 200, 20 and 2 p/μL, spotted on filter papers and air dried. Each reaction plate included a standard curve for parasite quantification. Positive samples were confirmed by an independent DNA extraction and a new round of qPCR by triplicates. Samples were considered positive with at least two positive wells in the confirmation reaction.

### Duffy genotype assessment

The sample size was calculated with a confidence level of 95%, error of 5% and an estimated prevalence of the Duffy negative genotype of 30% with a population census of 5,603 for all the sentinel sites. DNA extraction was performed from 200 μL of total blood samples collected in EDTA tubes, using the PureLink Genomic DNA Mini Kit (Invitrogen, USA) following manufacturer’s instructions. The Duffy allele genotyping was performed by qPCR using SYBR Green chemistry as described by Sousa *et al.* [21] previously standardized to screen volunteers for malaria clinical trials at Caucaseco Scientific Research Center [22]. In each plate, positive controls were included for genotypes FY*A/FY*A, FY*A/FY*B, FY*A/FY*B^ES^, and FY*B^ES^/FY*B^ES^.

### Data analysis

#### Data entry

Study data were collected and managed using REDCap electronic data capture tools hosted at Caucaseco Scientific Research Center [23]. REDCap (Research Electronic Data Capture) is a secure, web-based application designed to support data capture for research studies, providing: 1) an intuitive interface for validated data entry; 2) audit trails for tracking data manipulation and export procedures; 3) automated export procedures for seamless data downloads to common statistical packages; and, 4) procedures for importing data from external sources. Information was captured in the field in paper-based case report forms (CRF). Data were digitized using REDCap (version 4.1) and imported into MATLAB (version 2011b) for analysis.

### Data quality assurance

The data quality assurance (QA) procedure consisted of setting a quality control (QC) sample of size q less than the total number p of all case report forms (CRFs) for each location. The maximum permissible error for this study was set to 1%.

### Statistical analysis

Data analysis was performed using MATLAB (version 2011b). Using descriptive statistics, the general characteristics of individuals admitted to the study were established.

### Ethics statement

This study was carried out in accordance with institutional guidelines. The protocol was previously reviewed and approved by the institutional review board (IRB) of Caucaseco Scientific Research Center (CECIV, Cali-Colombia). Written informed consent (IC) was obtained from each volunteer at enrolment. Parents were asked to consent for their child to take part in the study. Information obtained from the participants was managed on principles of confidentiality. Immediately after blood donation, malaria-positive volunteers were provided with the anti-malarial treatment recommended by the Colombian Ministry of Health.

## Results

### Demographic features

Between October 2011 and January 2012 a total of 1,169 subjects (57.7% female) were enrolled in three malaria-endemic sites. A total of 272 volunteers were recruited in Tierralta, 460 in Tumaco and 437 in Buenaventura (Table 1). The mean age was 27 years (median 21 years; interquartile range (IQR) 10-41 years) for all volunteers, children ≤15 years of age (39.7%) were the main population. Of the entire study population, 579 subjects (50.3%) self-reported to have experienced previous lifetime malaria episodes with a median of two previous episodes.

**Table 1:** **Demographics of study populations**

		**Buenaventura N = 437**	**Tierralta N = 272**	**Tumaco N = 460**	**Total N = 1,169 **
		**% [n]**	**% [n]**	**% [n]**	**% [n]**
**Age (years)**	0-4	12.7 [54]	11.3 [30]	4.4 [20]	9 [104]
	5-14	26.8 [113]	33.8 [90]	239 [109]	27.3 [312]
	15-30	27.3 [115]	24.8 [66]	29.4 [134]	27.6 [315]
	>30	33.2 [140]	30.0 [80]	42.2 [192]	36.0 [412]
**Sex**	Female	57.7 [252]	51.8 [141]	60.4 [278]	57.7 [674]
**Previous episodes of malaria**	Yes	56.7 [242]	57.0 [154]	40.3 [183]	50.0 [579]

### Prevalence of *Plasmodium vivax* and *Plasmodium falciparum* infections

**Figure 1:**
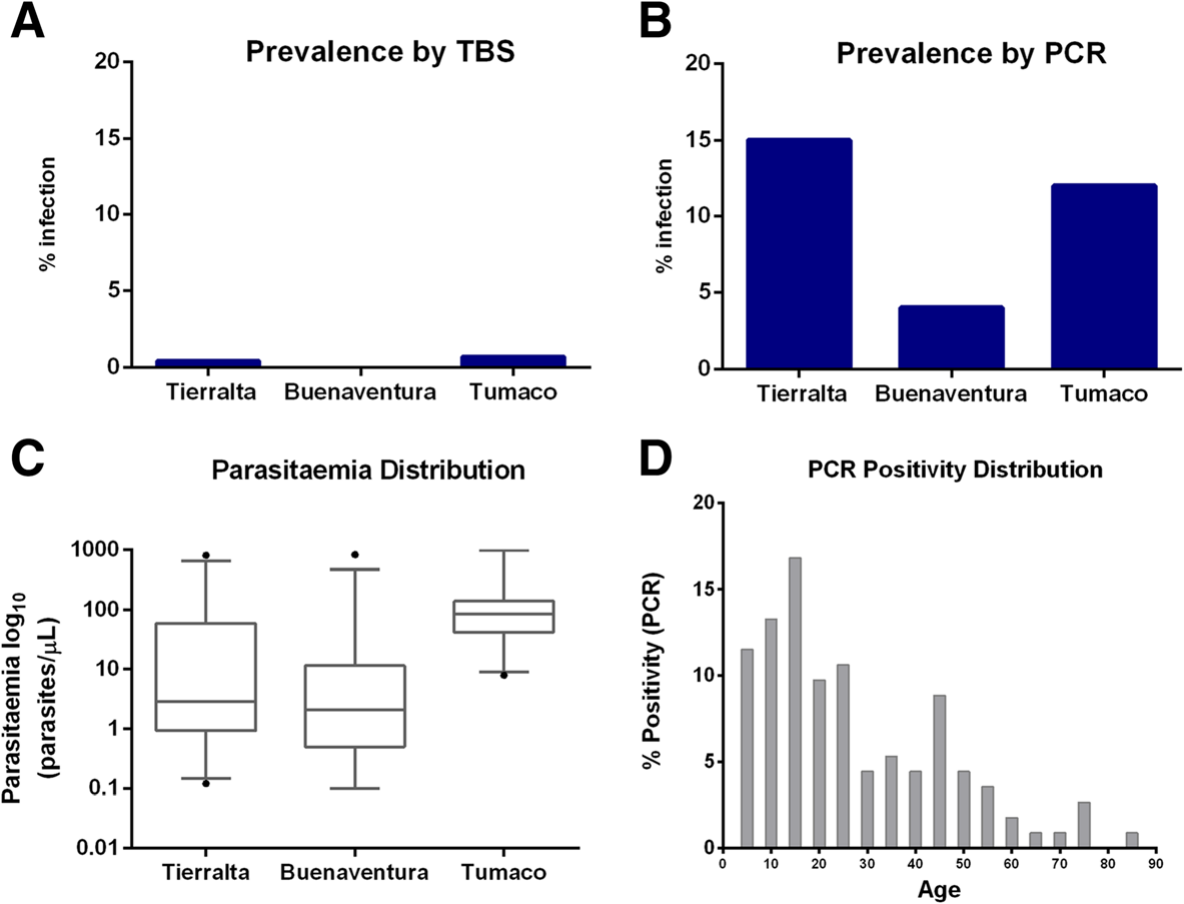
Malaria prevalence in the study sites. **(A)** Prevalence by TBS; **(B)** prevalence by qPCR; **(C)** parasitaemia levels in the study areas; **(D)** age stratification of qPCR-positive volunteers.

**Figure 2:**
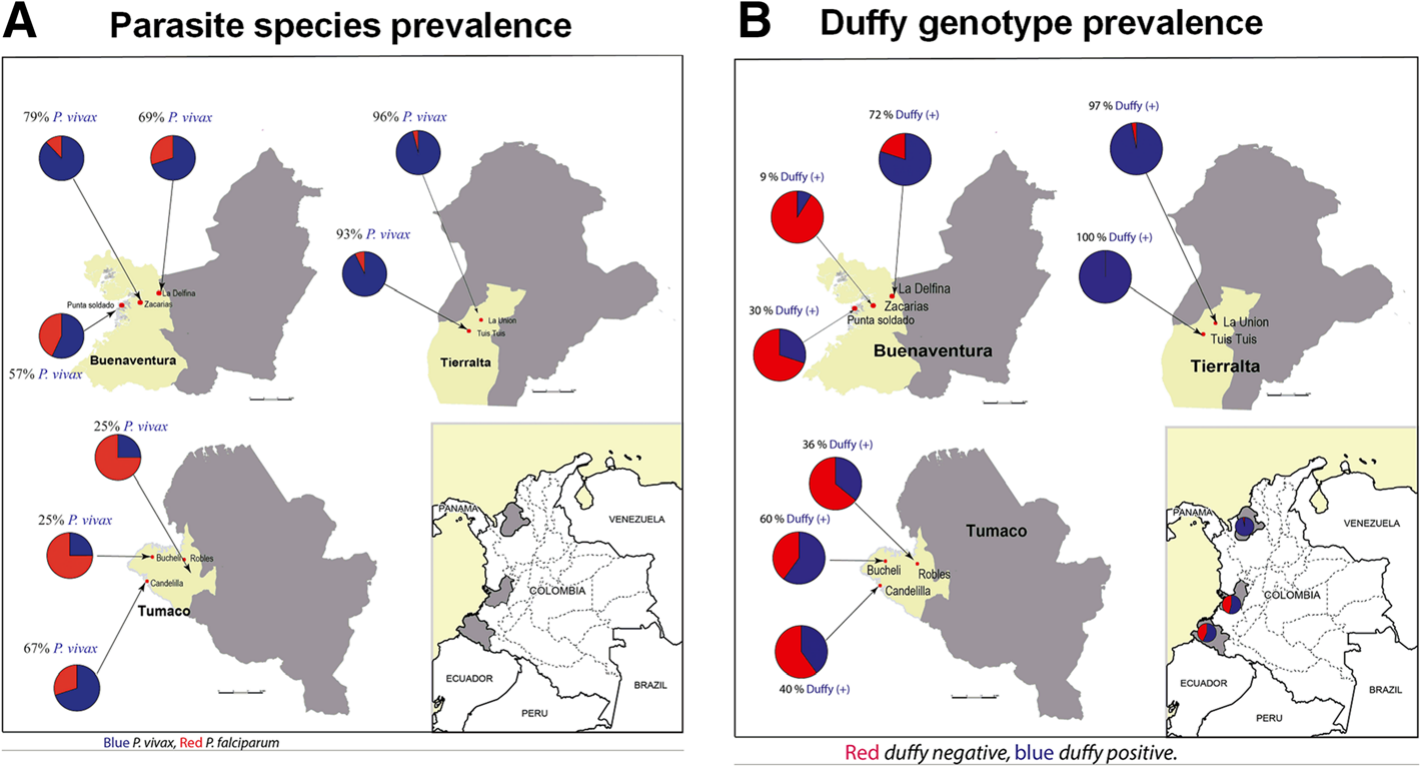
Parasite species and Duffy genotype distribution. **(A)** Parasite species distribution is shown for *P. vivax* (blue bars) and *P. falciparum* (red bars); **(B)** distribution of Duffy negative (red) and positive (blue) for each site within each region analysed is shown as a pie chart.

The overall malaria prevalence assessed in the three study sites by thick blood smear (TBS) was 0.3% (4/1,169). The prevalences in Tierralta and Tumaco were 0.4 (1/272) and 0.7% (3/460), respectively, whereas in Buenaventura no positives slides were detected (Figure 1A). However, by using qPCR an overall prevalence of 9.7% (113/1,169) was detected, over 26 times more cases than those detected by TBS. The positivity rate varied from 3 to 20%, indicating a large variability among the sentinel sites (Table 2). Among the three study regions, the highest positivity rate was in Tierralta (15%; 40/272), followed by Tumaco (4%; 20/460) and Buenaventura (12%; 53/437) (Figure 1B). The mean parasitaemia (parasites/μL) in Tierralta determined by qPCR was 56.74 (1-813), whereas in Buenaventura it was 60.7 (1-1,673) and in Tumaco it was 143.9 (8-1,021) (Figure 1C). Among the qPCR-positive volunteers, 0.4% (n = 5) presented symptoms, and the highest positivity rate was observed among the youngest people (0-15 years old) with a peak in habitants between 40-50 years old (Figure 1D). The overall parasite distribution was 73.5% *P. vivax*, 22.1% *P. falciparum* and 4.4% mixed infections. The *P. vivax* prevalence in Tierralta, Buenaventura and Tumaco was 93, 66 and 50%, respectively (Figure 2A). The prevalence in each sentinel site is shown in Table 2 shows the number of positive qPCRs for detection of either *P. vivax* and *P. falciparum* from all samples or only asymptomatic individuals as indicated in the two corresponding qPCR(+) columns; TBS shows the number of positive samples with parasitaemia detected by microscopy (thick blood smear); Samples positive for the presence of P. vivax by qPCR are shown in the indicated column; FY(+) indicates the % of people presenting Duffy positive genotype.

**Table 2:** **Parasite and Duffy genotype prevalences**

	**Sites**	**qPCR+ (%)**	**qPCR+ (%) symptomatics**	**TBS+ (%)**	***P. vivax***	**FY (+) (%)**
**Buenaventura**	Punta Soldado	23/145 (16%)	0/23 (0%)	0/145 (0%)	13/25 (57%)	11/35 (30%)
	Zacarías	14/163 (9%)	2/14 (14%)	0/163 (0%)	11/14 (79%)	3/35 (9%)
	La Delfina	16/129 (12%)	0/16 (0%)	0/129 (0%)	11/16 (69%)	26/36 (72%)
	**Total**	**53/437 (12%)**	**2/53 (4%)**	**0/437 (0%)**	**35/53 (66%)**	**47/106 (44%)**
**Tierralta**	Tuis Tuis	15/146 (10%)	1/15 (7%)	0/146 (0%)	14/15 (93%)	39/39 (100%)
	La Unión	25/146 (20%)	0/25 (0%)	1/146 (1%)	24/25 (96%)	58/60 (97%)
	**Total**	**40/272 (15%)**	**1/40 (3%)**	**1/272 (0.4%)**	**38/40 (95%)**	**97/99 (98%)**
**Tumaco**	Robles	4/150 (3%)	0/4 (0%)	1/150 (1%)	1/4 (25%)	12/33 (36%)
	Candelilla	12/160 (8%)	0/12 (0%)	2/160 (1%)	8/12 (67%)	14/35 (40%)
	Bucheli	4/150 (3%)	2/4 (50%)	0/150 (0%)	1/4 (25%)	18/30 (60%)
	**Total**	**20/460 (4%)**	**2/20 (10%)**	**3/460 (0.6%)**	**10/20 (50%)**	**44/98 (46%)**
		**113/1169 (10%)**	**5/113 (0.4%)**	**4/1169 (0.3%)**	**83/113 (73%)**	**118/303 (62%)**

### Duffy genotype distribution

Of the 302 analysed samples, FY*B^*ES*^/FY*B^*ES*^ was the most frequent genotype (38.1%) in Colombia and FY*B/FY*B the least frequent (6.6%), based on the study population. However Duffy genotype distribution varied considerably among the three malaria-endemic regions. In Tierralta the most common genotype was FY*A/FY*B with 29.3%, followed by FY*A/FY*A (24.2%), FY*B/FY*B^*ES*^ (18.2%), FY*B/FY*B (15.2%), FY*A/FY*B^*ES*^ (10.1%), and finally FY*B^*ES*^/FY*B^*ES*^ with 3%. Contrasting results were found in Tumaco and Buenaventura, which are located on the Pacific coast, with a higher percentage of black African people than the Atlantic region. In Tumaco the most common genotype was FY*B^*ES*^/FY*B^*ES*^ with 53.6%, followed by FY*A/FY*B^*ES*^ (21.6%), FY*B/FY*B*^ES^ (13.4%), FY*A/FY*B (5.2%), FY*A/FY*A (4.1%), and finally FY*B/FY*B (2.1%). A similar pattern was found en Buenaventura, being the most common FY*B^*ES*^/FY*B^*ES*^ with 56.6%, followed by FY*A/FY*B^*ES*^ (14.2%), FY*B/FY*B^*ES*^ (10.4%), FY*A/FY*A (8.5%), FY*A/FY*B (7.5%), and the least common FY*B/FY*B with 2.8%. Table 2 shows the Duffy prevalence by sentinel site. The parasites geographic distribution correlates with the Duffy genotype distribution (Figure 2B).

## Discussion

This study shows that in low transmissions settings of Colombia there is a considerable amount of circulating parasites at low parasitaemias which are not detected by the current methods employed by the malaria control programme. The prevalence of sub-microscopic infections varies largely between 3 to 20%, with a higher rate in regions where *P. vivax* is the predominant species. The prevalence of malaria sub-microscopic infections in low-transmission areas could be dependent on the detection method used. This finding may explain the maintenance or persistence of residual transmission foci due to the existence of asymptomatic human reservoirs with parasitaemia that are not regularly detected. Undetected asymptomatic infections do not receive proper treatment, contributing to maintaining the transmission cycle to *Anopheles* vectors and therefore malaria transmission. Additionally, our results demonstrate the effectiveness of qPCR to detect these parasitaemias. The availability of an efficient and effective technique for detecting these infections would be a key factor for decision-making. Two other studies in Colombia showed the presence of asymptomatic malaria [16,17], however no estimation of sub-microscopic infections was provided due to the lack of a quantitative molecular method.

Accurate parasite prevalence determination is key for monitoring malaria interventions. In this study, we provide first evidence showing submicroscopic parasitaemia levels among apparently healthy volunteers in endemic regions of Colombia. Several studies have reported detection of infection by molecular methods. A study in Rondônia, Brazil, showed that PCR was 6–7 times more efficient than microscopy in detecting malaria infections [24]. A previous study in Colombia using end point PCR showed that molecular tools detected 61% more infections than microscopy [16]. These results support the need for implementing more sensitive diagnosis techniques in order to detect these malaria reservoirs.

The Duffy glycoprotein antigen on red blood cells is a receptor that binds the malaria parasite *P. vivax*, and red blood cells that lack these antigens are resistant to invasion by this parasite but can still be invaded by *P. falciparum* [25]. In malaria-endemic regions, the Duffy genotype distribution affects the proportion of malaria infections due to Duffy-negative population that are refractory to *P. vivax* infections. On the southern Pacific coast of Colombia (Buenaventura and Tumaco), the prevalence of the Duffy-negative genotype was 56%, compared to the northern region (Tierralta) where the corresponding percentage was around 3%. On the other hand, the frequency of Duffy-positive genotype was higher in Tierralta than in Tumaco and Buenventura, these two areas being endemic for *P. falciparum* and Tierralta for *P. vivax.* The parasite species distribution correlates with the Duffy genotype prevalence, as *P. vivax* infection detected among the Duffy positive individuals was five times higher than *P. falciparum.* The distribution of the negative Duffy genotype on the Pacific coast is consistent with the predominant Afro-descendants and the *Mestizo* population migration patterns in Colombia. Furthermore, the very low frequency of Duffy negative factor found in the North coast population could be due to a higher percentage of interbreeding with indigenous populations and a low migration pattern of Afro-descendants.

Because malaria prevalence officially reported by Pan American Health Organization/World Health Organization (PAHO/WHO) is based on official country data, it is significantly underestimated in the region [24,26,27]. Sub-microscopic malaria infections have been increasingly reported in malaria-endemic areas, especially in places where malaria transmission intensities are relatively low and where malaria elimination is being targeted [28]. Asymptomatic infections that remain undetected and untreated contribute a major source of gametocytes for local mosquito vectors, resulting in malaria transmission.

Whereas there is general agreement about sub-reporting and other critical logistic aspects related to delivering health services, the notion of sub-microscopic malaria that results in undetectable transmission is not recognized as of local or regional importance. Thus, biological components of transmission need to be considered as a crucial part of any research malaria agenda that aims to support elimination. Additionally, targeting sub-microscopic carriers will be a challenge for malaria control programmes. Possible approaches to include sub-microscopic carriers in treatment campaigns include mass screening and treatment of all parasite carriers (MSAT) and mass treatment without prior screening for parasitaemia, i.e., mass drug administration (MDA). MSAT depends on the availability of very sensitive tools that could be used under field conditions. A very low parasitaemia can be detected by molecular methods such as qPCR; however, these techniques require considerable training and are restricted to reference laboratories. Diagnostic tools, such as the Loop mediated isothermal amplification (LAMP), which are capable of detecting very low parasite densities (one parasite/μL) and are reliable under field conditions could be important for active case detection to accelerate malaria elimination [29]. LAMP has been tested in Colombia under field conditions with outstanding results [19]. However, cost-effectiveness studies and proof of concept are needed in order to include this technique in malaria control programmes. An alternative approach from this study is to target MDA to those households with the highest prevalence of infection detected by molecular techniques. This approach could increase the proportion of true positives who would be treated [30].

Microscopy continues to be the gold standard method both for malaria routine diagnosis and elimination [2], however, the high occurrence of asymptomatic and sub-microscopic infections only detectable by more sensitive molecular techniques underscores the urgent need for implementation of targeted surveillance strategies and response activities in malaria elimination campaigns. These findings have a deep impact on compliance with the goals and in the evaluation of policies, plans, programmes, and projects aimed to achieve malaria elimination in Colombia. Indeed, in the Colombian Public Health Plan 2012-2021, the malaria elimination in urban/peri-urban and hypo-endemic settings was settled as a main goal. This political change represents an opportunity to put into operation the new strategies presented in this work to improve the operational strategies required for progress towards malaria elimination.

## Conclusion

The presence of sub-microscopic malaria infections in different regions in Colombia shown here, especially in areas of low transmission intensity, underscores the importance of conducting active case surveillance to more accurately determine malaria prevalence in endemic regions, as well as to eliminate malaria reservoirs. There is a need to use techniques more sensitive than microscopy-based TBS for detection of these sub-microscopic infections.
